# Sexually transmitted infections among HIV-infected and HIV-uninfected women in the Tapajós region, Amazon, Brazil: Self-collected vs. clinician-collected samples

**DOI:** 10.1371/journal.pone.0215001

**Published:** 2019-04-23

**Authors:** Luana L. S. Rodrigues, Justin Hardick, Alcina F. Nicol, Mariza G. Morgado, Katrini G. Martinelli, Vanessa S. de Paula, José H. Pilotto, Charlotte A. Gaydos

**Affiliations:** 1 Laboratório de AIDS e Imunologia Molecular, Instituto Oswaldo Cruz, Fundação Oswaldo Cruz, Rio de Janeiro, Brazil; 2 Instituto de Saúde Coletiva, Universidade Federal do Oeste do Pará, Santarém, Pará, Brazil; 3 Division of Infectious Diseases, Johns Hopkins University School of Medicine, Baltimore, Maryland, United States of America; 4 Instituto Nacional de Infectologia Evandro Chagas, Fundação Oswaldo Cruz, Rio de Janeiro, Brazil; 5 Departamento de Medicina Social, Universidade Federal do Espírito Santo, Vitória, Espírito Santo, Brazil; 6 Laboratório de Virologia Molecular, Instituto Oswaldo Cruz, Fundação Oswaldo Cruz, Rio de Janeiro, Brazil; London School of Hygiene and Tropical Medicine, UNITED KINGDOM

## Abstract

The anogenital prevalence of sexually transmitted infections (STIs) and the use of cervico-vaginal self-collected vs. clinician-collected samples were evaluated for the diagnosis of human immunodeficiency virus (HIV)-infected and HIV-uninfected women in the Tapajós region, Amazon, Brazil. We recruited 153 women for a cross-sectional study (112 HIV-uninfected and 41 HIV-infected) who sought health services. Anal and cervical scrapings and cervico-vaginal self-collection samples were collected. Real-time polymerase chain reaction methods were used for *Chlamydia trachomatis*, *Neisseria gonorrhoeae*, *Trichomonas vaginalis* and *Mycoplasma genitalium*. A syphilis test was also performed. Risk factors for STIs were identified by multivariate analysis. The overall prevalence of STIs was 30.4% (34/112) in HIV-uninfected women and 24.4% (10/41) in HIV-infected women. Anogenital *Chlamydia trachomatis* infection was the most prevalent in both groups of women (20.5% vs 19.5%). There was significant agreement for each STI between self-collected and clinician-collected samples: 91.7%, kappa 0.67, 95% confidence interval (CI) 0.49–0.85 for *Chlamydia trachomatis*; 99.2%, kappa 0.85, 95% CI 0.57–1.00 for *Neisseria gonorrhoeae*; 97.7%, kappa 0.39, 95% CI -0.16–0.94 for *Trichomonas vaginalis*; and 94.7%, kappa 0.51, 95% CI 0.20–0.82 for *Mycoplasma genitalium*. Women with human papillomavirus had coinfection or multiple infections with other STIs. Risk factors for STIs were being ≤ 25 years old, being employed or a student, reporting a history of STI and having a positive HPV test. A high prevalence of STIs in women in the Tapajós region was found. Cervico-vaginal self-collection is a useful tool for STI screening and can be used in prevention control programs in low-resource settings, such as in northern Brazil.

## Introduction

The World Health Organization (WHO) estimates that among adults aged 15–49 years there are 357 million new infections each year of either *Chlamydia trachomatis* (CT), *Neisseria gonorrhoeae* (NG), *Trichomonas vaginalis* (TV) or syphilis. This represents approximately one million new infections every day [1]. Most of these infections are treatable with currently available antibiotics; however, antimicrobial resistance is a growing threat for NG, syphilis and *Mycoplasma genitalium* (MG) [2]. If untreated, STIs can cause several serious complications and increased risks of human immunodeficiency virus 1 (HIV-1) acquisition and transmission and cancer [3].

Very few population-based studies of STI prevalence exist for low- and middle-income countries [3]. In Brazil, the epidemiological surveillance data of STIs provided by the Ministry of Health are essentially restricted to HIV/AIDS, hepatitis and syphilis [4]. Some isolated studies in Brazil show a varied prevalence of STIs that depends on the infectious agent, population approached, sample size and laboratory techniques used [5,6]. In Brazil, 5.3% of women self-reported having had an STI in 2013. Women appear to carry a greater burden of STIs and self-reported STIs that were associated with increasing age, decreasing socioeconomic status, current or previous drug use, sex with a casual partner in the last 12 months, sex with the same sex partner, nonindigenous status and one or more previous HIV tests [7]. In the Amazon, accessibility, measured as the travel time via local transportation to a public health unit, remains a challenge for health care access, as well as for scientific research in these places [8], and this may be why there are very few studies on STIs in northern Brazil.

**Fig 1:**
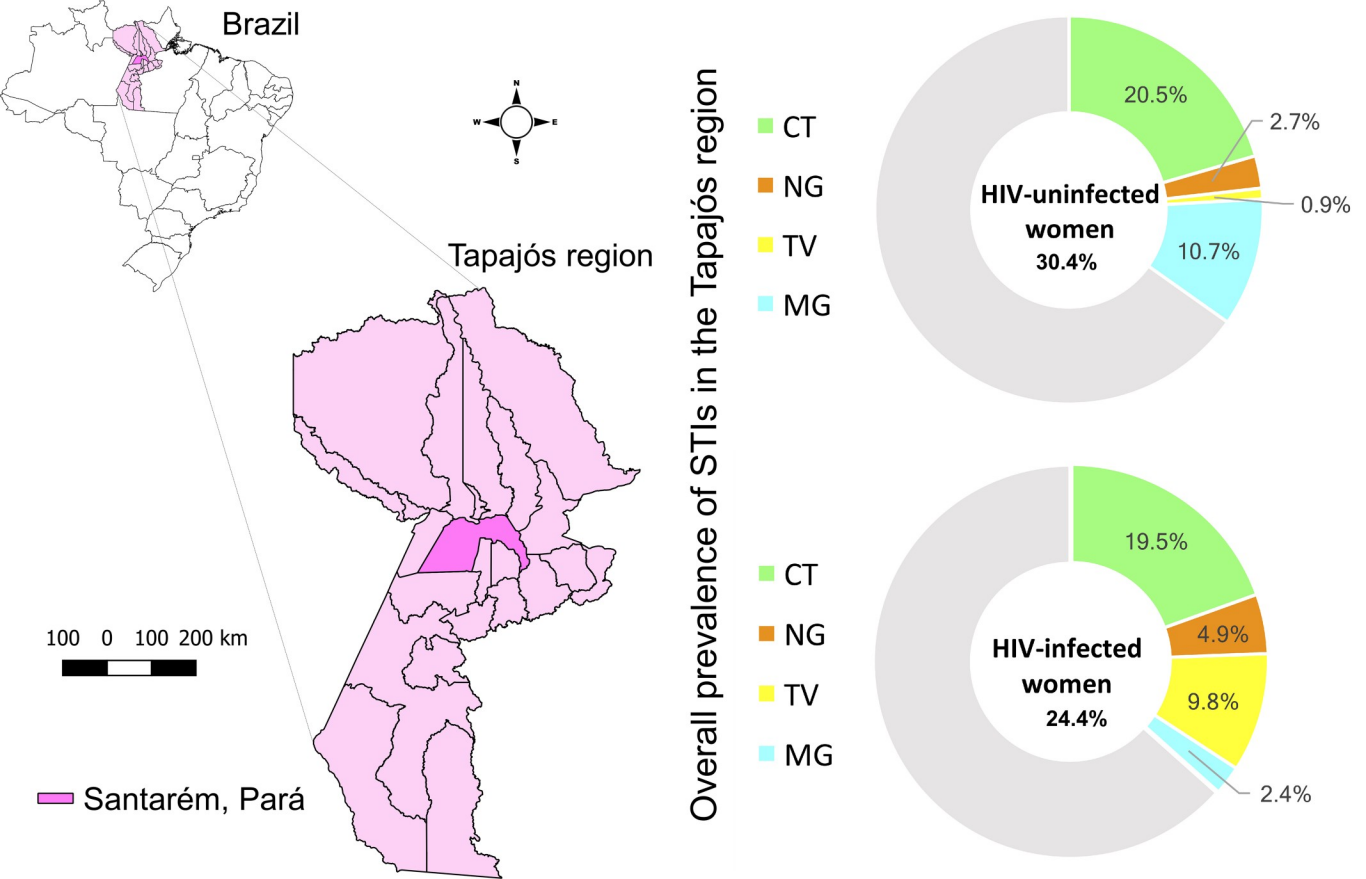
Representative map of the Tapajós region in northern Brazil. Highlight for Santarém and the 22 neighboring municipalities and the overall prevalence of STIs.

The Tapajós region is located in northern Brazil and comprises two meso-regions of the state of Pará, namely, the Lower Amazon and the Southwest (Fig 1). The main socioeconomic center is Santarém, which supports health services for 22 neighboring municipalities [9,10]; however, travel to Santarém for health services can be challenging and difficult [10]. Increasing knowledge regarding the epidemiology of CT, NG, TV and MG infection in women living in the Tapajós region is essential to provide surveillance data, a key element in the control and management of STIs. Moreover, the advantages of cervico-vaginal self-collection compared to clinician-collected samples include added privacy, convenience and time savings (since a provider appointment is not always necessary). Expanding STI testing options, especially in populations at risk [11], such as those who live in the Tapajós region.

The aim of this study was to determine the anogenital prevalence of STIs and to evaluate the use of cervico-vaginal self-collection versus clinician-collection samples for STI diagnosis in HIV-infected and HIV-uninfected women living in the Tapajós region, Amazon, Brazil.

## Materials and methods

### Study design and population

The cross-sectional study was conducted from August 2015 to August 2016 and included nonindigenous, HIV-infected and HIV-uninfected women living in the Tapajós region. A total of 186 women were invited to participate in the study, and 153 of those accepted and were eligible to enter the study. To ensure that the study population was representative, the participants were recruited from nine public health units strategically covering the central, semiperipheral and peripheral points of Santarém. The population of other municipalities search for health care in public health units in the outskirts of town, while the population of Santarém search for health care in public health units downtown. The accessibility and infrastructure of the public health units that had health professionals prepared to perform the sample collections were also considered. We also invited all HIV-infected women at the Santarém Counseling and Testing Center, the only referral center for the care and follow-up of people living with HIV in this region, to participate. The HIV-uninfected women voluntarily sought the public health unit to schedule pap smear collection and were then invited to participate in the study. HIV-infected women who either had an appointment with a healthcare provider without a scheduled gynecological examination or who went to the public health unit to get antiretrovirals were then invited to participate in the survey. The HIV diagnostic testing, HIV viral load determination, and CD4+ T-lymphocyte count were conducted according to the manufacturer’s instructions in the published protocol [12].

### Inclusion and exclusion criteria

The inclusion criteria were being 18 years of age or older (or consent from the legal guardian if the age was less than 18 years), sexually active and able to sign an informed consent form. The exclusion criteria were samples considered unsatisfactory on the pap smear, pregnancy, and use of antimicrobials in the previous 15 days. All women who met all inclusion criteria were considered eligible to participate in the survey.

### Ethical issues and data collection

The study was approved by the Universidade do Estado do Pará Ethics Committee Board (UEPA, protocol no. 1.099.852) and the Instituto Oswaldo Cruz (IOC-FIOCRUZ, protocol no. 1.059.253) Ethics and Research Committees. All women agreed to participate in the study and signed an informed consent form in compliance with the Brazilian human ethical guidelines. This study was supported by a previous study in which we evaluated the acceptability of the cervico-vaginal self-collection, and HIV and HPV tests and sociodemographic data are fully described elsewhere [12]. Participation in this study included the collection of sociodemographic and clinical data using a standardized epidemiological form followed by the collection of specimens. All data are presented in the Tables and Figs. Detailed dataset can be available under request.

### Specimen collection

Trained health professionals of the public health unit collected samples with a brush using anal scraping techniques. Similarly, cervical scrapings were collected with an endocervical brush during the pap smear, and women who agreed to perform the cervico-vaginal self-collection received the collection instructions and an individual collection kit. The self-collection was performed immediately after the pap smear in another office where the woman was alone, and at the end of the procedure, the sample was given to the provider to be labeled/identified and stored [12]. The anal and cervical scrapings were clinician-collected samples, and we compared cervical scrapings with the cervico-vaginal self-collection samples.

All collected specimens from scraping and self-collection were immediately stored in ThinPrep Pap Test (Hologic, Inc., San Diego, California, USA) at 5°C and transported to the Universidade Federal do Oeste do Pará in Santarém, Pará, for storage and, afterwards, were shipped to the Instituto Oswaldo Cruz, Fundação Oswaldo Cruz in Rio de Janeiro, Brazil. The transportation in Brazil was performed in appropriate polystyrene foam-insulated dry ice boxes with strict temperature monitoring and replenishment of dry ice on every shipment of the blood samples (-20°C to -80°C). All scraping and self-collection DNA samples were transported later to Johns Hopkins University in Baltimore, Maryland, United States of America, in appropriate packaging with strict temperature monitoring (-20°C to -80°C) and replacement of dry ice. All flight transport procedures in Brazil and the USA followed the International Air Transport Association (IATA) international requests for biological materials and were performed by a specialized commercial company (World Courier).

### DNA extraction and STI molecular detection

DNA extraction from the samples was performed with the QIAamp DNA Mini Kit (Qiagen, Valencia, California, USA) and quantified by spectrophotometry using a NanoDrop (ND 1000, Fisher Scientific, Wilmington, Delaware, USA).

**Fig 2:**
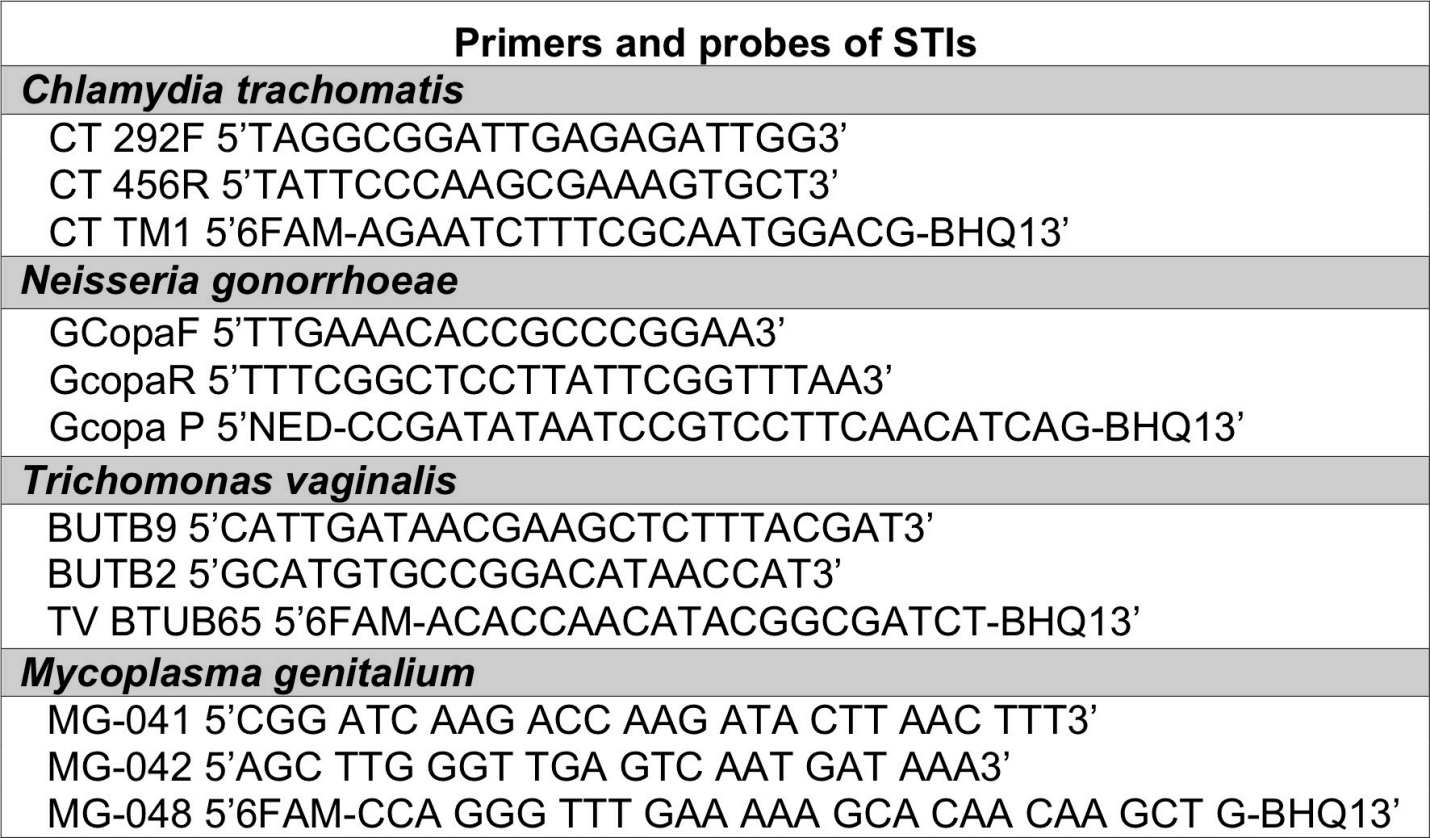
Primers and probes used to detect each STI.

A duplex real-time PCR was performed for CT and NG, which was validated at the International Chlamydia Research Laboratory at Johns Hopkins University, utilizing primers and probes from an unpublished assay with sensitivity and specificity of 93.8% and 99.5% for CT and 100% and 100% for NG, respectively, that was validated by comparison with the Roche Amplicor and Hologic Aptima Combo 2 assays. The NG primers were adapted from a previously published protocol [13]. Real-time PCR was performed to detect TV with 90.1% sensitivity and 100% specificity [14], and real-time pdhD PCR was performed to detect MG with 80% sensitivity and 95% specificity [15]. The primer and probe sequences used to detect CT, NG, TV, and MG are shown below (Fig 2).

All reactions included 40 μl of master mix (25 μl Taqman Fast Advance Master Mix (2X), 2 μl MgCl_2_ (25 mM), 2 μl each of the primers and probes (10 μM) and completed with dH_2_O) and 10 μl of sample DNA to a final volume of 50 μl of PCR reaction mixture. All reactions were performed in a QuantStudio 12K Flex Real-Time PCR System (Applied BioSystems by Life Technologies, Carlsbad, California, USA), and the parameters were 95°C for 20 s and 40 cycles of 95°C for 20 s and 60°C for 1 min.

### HPV detection and genotyping

A nested PCR was performed to increase the specificity of HPV DNA detection, with PGMY09 and PGMY11 primers for the first round and GP5+ and GP6+ primers for the second round, as previously described [16]. HPV genotyping was performed according to a previously published protocol [12].

### Syphilis test

The Alere Determine^TM^ Syphilis TP test (Delaware, USA) was used as a point-of-care screening test to detect antibodies to *Treponema pallidum* (TP) in the serum of HIV-infected women; the test has 100% sensitivity and 100% specificity, according to the manufacturer’s instructions [17].

### Statistical analysis

Descriptive statistics of the qualitative variables were determined by frequency distribution and the quantitative variables by medians and interquartile ranges (IQR) or means and standard deviation (SD). The chi-square test was used to compare the proportions between the groups for categorical variables and the Mann-Whitney U test for continuous variables, both at 95% CI and *P*-value ≤ 0.05. For risk factors, the univariate analysis and odds ratio (OR) were calculated, considering the *P*-value ≤ 0.20. In the multivariable model, only the variables with a *P*-value ≤ 0.05 remained in the model, and the adjusted odds ratio (aOR) was calculated. Spearman's ρ test was performed to evaluate the linear relationship between the CD4+ T-cell count and STIs. We considered 39 copies/ml for the HIV viral load below the limit of detection (40 copies/ml) for statistical purposes. The positive agreement between cervico-vaginal self-collection and cervical scraping was calculated using the Kappa test with 95% CI and *P-*value ≤ 0.05. All statistical analyses were performed using Statistical Package for Social Sciences (SPSS) software version 19.0 (IBM Corp., Armonk, USA).

## Results

### Sociodemographic characteristics of the women participants

A total of 153 women agreed to participate in the study, and overall, they provided 439 samples. Of these, 306 were collected by health professionals using anal and cervical scraping techniques, and 133 were self-collected by participants. The participants were split into two groups: HIV-uninfected women (n = 112) and HIV-infected women (n = 41). Overall, 53.1% had three or more pregnancies, 43.8% did not use hormonal contraception, 68.4% had her first sexual intercourse at or before 17 years of age, 54.7% reported four or more sexual partners in her life, 85.2% had pap smears regularly, 58.6% practiced anal sex, only 16.4% used condoms, and 30.5% reported a history of STIs. Additionally, 22.2% had abnormal pap smears, 49.7% had HPV DNA detected in cervical scrapings, and 86.9% performed the cervico-vaginal self-collection (Table 1). Some results have been reported previously in [12].

**Table 1:** Epidemiological variables of HIV-uninfected and HIV-infected women.

Variables		HIV-uninfected n = 112 (%)	HIV-infected n = 41 (%)	Total n = 153 (%)	Chi-square (*P*-value)
**Age (years)** (n = 153)					0.506
	≤20	08 (7.1)	05 (12.2)	13 (8.5)	
	21–30	36 (32.1)	08 (19.5)	44 (28.8)	
	31–40	26 (23.2)	12 (29.3)	38 (24.8)	
	41–50	23 (20.5)	10 (24.4)	33 (21.6)	
	>50	19 (17.0)	06 (14.6)	25 (16.3)	
**Employment status** (n = 153)					0.585
	Unemployed	60 (53.6)	24 (58.5)	84 (54.9)	
	Employed or student	52 (46.4)	17 (41.5)	69 (45.1)	
**Marital status** (n = 150)					0.006
	Single	36 (32.7)	23 (57.5)	59 (39.3)	
	Married/living together	74 (67.3)	17 (42.5)	91 (60.7)	
**Education (years)** (n = 152)					0.146
	Up to 14	34 (30.6)	18 (43.9)	52 (34.2)	
	15–17	57 (51.4)	20 (48.8)	77 (50.7)	
	18+	20 (18.0)	03 (7.3)	23 (15.1)	
**City of birth** (n = 153)					0.191
	Santarém, Pará	78 (69.6)	25 (61.0)	103 (67.3)	
	Other municipalities in the Tapajós region	16 (14.3)	11 (26.8)	27 (17.6)	
	Outside the Tapajós region	18 (16.1)	05 (12.2)	23 (15.0)	
**City of residence** (n = 152)					0.005
	Santarém, Pará	111 (99.1)	35 (87.5)	146 (96.1)	
	Other municipalities in the Tapajós region	01 (0.9)	05 (12.5)	06 (3.9)	
**Number of pregnancies** (n = 145)					0.595
	None	14 (13.0)	03 (8.1)	17 (11.7)	
	Up to 2	39 (36.1)	12 (32.4)	51 (35.2)	
	3+	55 (50.9)	22 (59.5)	77 (53.1)	
**Hormonal contraception** (n = 153)					0.004
	Yes	39 (34.8)	07 (17.1)	46 (30.1)	
	No	40 (35.7)	27 (65.9)	67 (43.8)	
	No information	33 (29.5)	07 (17.1)	40 (26.1)	
**History of cancer** (n = 132)					0.167
	Yes	15 (16.5)	11 (26.8)	26 (19.7)	
	No	76 (83.5)	30 (73.2)	106 (80.3)	
**Alcohol use** (n = 152)					0.447
	Yes	32 (28.6)	14 (35.0)	46 (30.3)	
	No	80 (71.4)	26 (65.0)	106 (69.7)	
**Tobacco use** (n = 152)					0.973
	Yes	16 (14.4)	06 (14.6)	22 (14.5)	
	No	95 (85.6)	35 (85.4)	130 (85.5)	
**Illicit drug use** (n = 151)					0.124
	Yes	02 (1.8)	03 (7.3)	05 (3.3)	
	No	108 (98.2)	38 (92.7)	146 (96.7)	
**Age of first sexual intercourse (years)** (n = 152)					0.052
	Up to 17	71 (64.0)	33 (80.5)	104 (68.4)	
	18+	40 (36.0)	08 (19.5)	48 (31.6)	
**Number of sex partners** (n = 150)					0.012
	1–3	57 (51.4)	11 (28.2)	68 (45.3)	
	4+	54 (48.6)	28 (71.8)	82 (54.7)	
**Regular pap smear (**n = 135)					0.310
	Yes	82 (87.2)	33 (80.5)	115 (85.2)	
	No	12 (12.8)	08 (19.5)	20 (14.8)	
**Condoms use** (n = 152)					0.100
	Yes	13 (11.7)	12 (29.3)	25 (16.4)	
	No	98 (88.3)	29 (70.7)	127 (83.6)	
**Practiced anal sex** (n = 152)					0.457
	Yes	67 (60.4)	22 (53.7)	89 (58.6)	
	No	44 (39.6)	19 (46.3)	63 (41.4)	
**History of STI**^**a**^ (n = 151)					0.126
	No	81 (73.0)	24 (60.0)	105 (69.5)	
	Yes	30 (27.0)	16 (40.0)	46 (30.5)	
	Types of STI:				
	Anogenital warts	22 (19.6)	09 (22.0)	31 (20.3)	
	Herpes	05 (4.5)	01 (2.4)	06 (3.9)	
	Gonorrhea	01 (0.9)	01 (2.4)	02 (1.3)	
	Syphilis	01 (0.9)	04 (9.8)	05 (3.3)	
	Hepatitis B	01 (0.9)	0 (0.0)	01 (0.7)	
	No information	01 (0.9)	01 (2.4)	02 (1.3)	
**Pap smear result** (n = 153)					^b^
	Negative	104 (92.9)	15 (36.6)	119 (77.8)	
	Inflammation	08 (7.1)	21 (51.2)	29 (19.0)	
	LSIL	0 (0.0)	03 (7.3)	03 (2.0)	
	HSIL	0 (0.0)	02 (4.9)	02 (1.3)	
**HPV test result** (n = 153)					<0.0001
	Negative	75 (67.0)	02 (4.9)	77 (50.3)	
	Positive	37 (33.0)	39 (95.1)	76 (49.7)	
**Cervico-vaginal self-collection** (n = 153)					0.069
	Yes	94 (83.9)	39 (95.1)	133 (86.9)	
	No	18 (16.1)	02 (4.9)	20 (13.1)	
**HIV viral load** (n = 41)		^c^			-
	<40 copies/ml		31 (75.6)	-	
	≥40 copies/ml		10 (24.4)	-	
**CD4+ T-cell count** (N = 41)		^c^			-
	<200 cells/mm^3^		08 (19.5)	-	
	200–500 cells/mm^3^		20 (48.8)	-	
	>500 cells/mm^3^		13 (31.7)	-	

a The same person may have had more than one prior STI.

b Since the number of HIV-infected women in the Hepatitis B category was zero, the *P*-value could not be calculated. Since the number of HIV-uninfected women in the LSIL and HSIL categories was zero, the *P*-value could not be calculated.

c HIV-uninfected women did not perform CD4+ T-cell counts and HIV viral load testing.

LSIL: low-grade squamous intraepithelial lesion; HSIL: high-grade squamous intraepithelial lesion.

Some of the data in Table 1 have been previously presented in [12].

### Overall anogenital prevalence of STIs according to the sample type

A high prevalence of STIs was identified in the population studied, with occurrences in 30.4% (34/112) of HIV-uninfected and 24.4% (10/41) of HIV-infected women based on positivity in at least one of the three clinical sample types, although no statistically significant difference was observed (*P* = 0.470). Women diagnosed with cervical lesions and inflammation due to bacterial vaginosis were referred for medical care follow-up.

**Fig 3:**
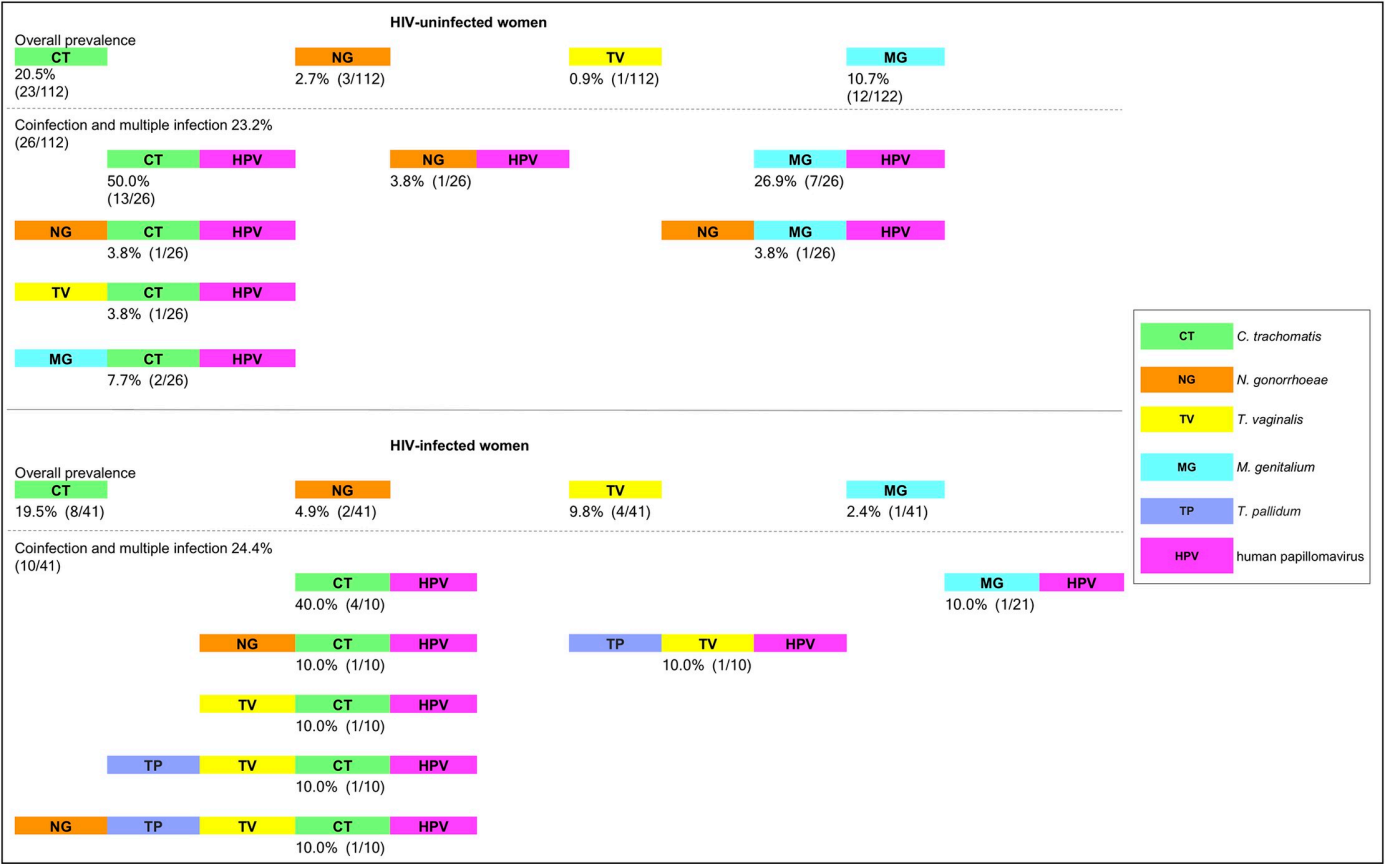
Overall prevalence of each STI and the percentage of coinfection and multiple infection. Coinfection is defined as a sample testing positive for two different microorganisms, for example, CT and HPV, while multiple infection is defined as a sample testing positive for three or more different microorganisms, for example, NG and CT and HPV. The number of HPV types was not used to define either coinfection or multiple infection.

HIV-uninfected women had the highest anogenital prevalences of CT (20.5%) and MG (10.7%), while in HIV-infected women, the prevalences for CT (19.5%) and TV (9.8%) were highest, based on positivity in at least one of the three types of clinical specimens (Fig 3). The STI prevalence according to the sample type among HIV-uninfected and HIV-infected women is shown in Table 2.

**Table 2:** Prevalence of STIs in cervico-vaginal self-collection, cervical scraping and anal scraping samples.

Sample type		CT		NG		TV		MG	
		n	% (95% CI)	N	% (95% CI)	n	% (95% CI)	n	% (95% CI)
**HIV-uninfected women (n = 112)**									
	Self-collection (n = 94)	13	13.8 (7.1–21.7)	2	2.1 (0.0–5.6)	1	1.1 (0.0–3.3)	9	9.6 (4.1–15.5)
	Cervical scraping (n = 112)	15	13.4 (7.5–20.4)	1	0.9 (0.0–2.9)	1	0.9 (0.0–2.9)	4	3.6 (0.9–7.6)
	Anal scraping (n = 112)	12	10.7 (5.3–16.8)	2	1.8 (0.0–4.5)	0	0	3	2.7 (0.0–6.1)
Total women*		23	20.5 (13.4–28.7)	3	2.7 (0.0–5.8)	1	0.9 (0.0–2.9)	12	10.7 (5.3–16.7)
**HIV-infected women (n = 41)**									
	Self-collection (n = 39)	6	15.4 (5.1–27.5)	2	5.1 (0.0–13.3)	1	2.6 (0.0–8.6)	1	2.6 (0.0–8.6)
	Cervical scraping (n = 41)	7	17.1 (6.3–30.3)	2	4.9 (0.0–12.5)	3	7.3 (0.0–16.2)	1	2.4 (0.0–8.1)
	Anal scraping (n = 41)	1	2.4 (0.0–8.1)	2	4.9 (0.0–12.5)	0	0	0	0
Total women*		8	19.5 (7.7–32.4)	2	4.9 (0.0–12.5)	4	9.8 (0.0–19.5)	1	2.4 (0.0–8.1)

Self-collection: Cervico-vaginal self-collection, CT: *Chlamydia trachomatis*, NG: *Neisseria gonorrhoeae*, TV: *Trichomonas vaginalis*, MG: *Mycoplasma genitalium*, CI: confidence interval.

* Total number women considering positivity in at least one of the three types of clinical specimens.

### Self-collected versus clinician-collected samples

The cervical scraping samples were used as clinician-collected samples for comparisons with cervico-vaginal self-collection using the Kappa test, with 95% CI, and *P* ≤ 0.05. There was significant agreement for each STI detected in the cervico-vaginal self-collection and cervical scraping groups. The comparisons of the CT DNA, NG DNA, TV DNA and MG DNA evaluations for both collection methods are shown in Table 3.

**Table 3:** Concordance between cervico-vaginal self-collection and cervical scraping collection calculated using the Kappa test.

STIs	CT	NG	TV	MG
**All participants (n = 133)**				
Agreement	91.7%	99.2%	97.7%	94.7%
Positive	122/133	132/133	130/133	126/133
kappa (95% CI)	0.67 (0.49–0.85)	0.85 (0.57–1.00)	0.39 (-0.16–0.94)	0.51 (0.20–0.82)
*P*-value	<0.0001	<0.0001	<0.0001	<0.0001
**HIV-uninfected (n = 94)**				
Agreement	91.5%	98.9%	100%	92.6%
Positive	86/94	93/94	94/94	87/94
kappa (95% CI)	0.64 (0.42–0.87)	0.66 (0.04–1.00)	1.00	0.43 (0.09–0.77)
*P*-value	<0.0001	<0.0001	<0,0001	<0.0001
**HIV-infected (n = 39)**				
Agreement	92.3%	100%	-	100%
Positive	36/39	39/39	-	39/39
kappa (95% CI)	0.72 (0.43–1.00)	1.00	-	1.00
*P*-value	<0.0001	<0.0001	0.814	<0.0001

CT: *Chlamydia trachomatis*, NG: *Neisseria gonorrhoeae*, TV: *Trichomonas vaginalis*, MG: *Mycoplasma genitalium*, CI: confidence interval.

It is important to note that substantial agreement (kappa> 0.60) was observed for comparisons of CT or NG DNA for all participants, HIV infected women, and HIV uninfected women. MG DNA showed substantial to moderate agreement among HIV-infected and non-HIV-infected women, respectively. The TV DNA presented the worst agreement for the two collection methods (Table 3).

### Coinfections and multiple infections and association with HPV in HIV-uninfected and HIV-infected women

The prevalence of each STI and the percentage of coinfections and multiple infections in both groups was 23.2% (26/112) in HIV-uninfected women and 24.4% (10/41) in HIV-infected women (Fig 3). No statistically significant differences were observed (*P* = 0.879). In Fig 3, when we described the prevalence of each STI (CT, NG, TV and MG DNA) and coinfections and multiple infections (STI-HPV and STIs-HPV), "coinfection" is defined when the woman was positive for two microorganisms of different classes, for example CT and HPV, while "multiple infection" is defined when the woman was positive for three or more microorganisms of different classes, for example NG and CT and HPV. In this way, the criterion of coinfections and multiple infections was based on the quantity and diversity of microorganisms that the woman had, independent of the number of HPV types she was infected with.

For the HIV-uninfected women, the coinfection rate by decreasing order of prevalence was 50.0% (13/26) for CT-HPV, 26.9% (7/26) for MG-HPV, 7.7% (2/26) for MG-CT-HPV, and 3.8% (1/26) for NG-HPV, for NG-CT-HPV, TV-CT-HPV and for NG-MG-HPV. The group of HIV-infected women had a higher diversity of STIs, and the seroprevalence of syphilis was 9.8% (4/41). Coinfections by decreasing order of prevalence were 40.0% (4/10) for CT-HPV, and 10.0% (1/21) for MG-HPV, for NG-CT-HPV, TV-CT-HPV, TV-HPV, TP-TV-CT-HPV and NG-TP-TV-CT-HPV (Fig 3).

HPV infection was detected in all cases of coinfection and multiple infections, and women with positive HPV DNA tests were compared with those with negative tests. Women with positive HPV tests had coinfection or multiple infections with other STIs, and a statistically significant difference was observed (*P* = 0.004).

Additionally, using sequencing, for the HIV-uninfected women with coinfections and multiple infections, 61.5% (16/26) had only one HPV type and 38.5% (10/26) had multiples HPV types; all HIV-infected women positive for any of the four STIs had multiples HPV types (100%; 10/10). HPV types and classification by high oncogenic risk identified per woman are detailed below (Table 4).

**Table 4:** Number and HPV types identified per woman with coinfection and multiple infections.

**HIV-uninfected women N = 26 (23.2%)**			
**Patient ID**	**Coinfection (STI-HPV)**	**Patient ID**	**Multiple infection (STIs-HPV)**
44	CT-hrHPV58	70	CT-NG-HPV6
88	CT-hrHPV16	85	CT-MG-HPV6-hrHPV16
120	CT-HPV34	12	CT-TV-hrHPV18-hrHPV45
47	CT-hrHPV16	106	CT-MG-hrHPV18-HPV54-HPV82
29	CT-HPV62	73	NG-MG-hrHPV16-hrHPV33-HPV53
42	CT-HPV83		
108	CT-hrHPV18		
74	NG-hrHPV33		
82	MG-HPV6		
92	MG-hrHPV31		
93	MG-HPV6		
109	MG-HPV83		
110	MG-hrHPV16		
65	MG-hrHPV16		
105	MG-hrHPV31		
16	CT-hrHPV33-HPV87		
21	CT-hrHPV16-HPV83		
41	CT-hrHPV45-HPV70		
86	CT-HPV6-hrHPV16		
103	CT-HPV72-HPV84		
111	CT-HPV54-HPV69		
**HIV-infected women n = 10 (24.4%)**			
**Patient ID**	**Multiple infection (STI-HPV)**	**Patient ID**	**Multiple infection (STIs-HPV)**
112	CT-HPV35-HPV40-HPV42- HPV44-hrHPV45-hrHPV56-hrHPV59-HPV61-HPV68-HPV69-HPV82	130	CT-TV-HPV11-hrHPV31-HPV35-HPV40-HPV42-hrHPV51-hrHPV58-HPV68-HPV73-HPV82-HPV83
118	CT-hrHPV33-HPV40-HPV42-HPV43-HPV44-hrHPV51-hrHPV56-hrHPV58-hrHPV59-HPV68-HPV70-HPV73-HPV82	116	CT-NG-HPV11-hrHPV16-hrHPV31-HPV53-HPV54-hrHPV51-HPV82
153	CT-HPV42-HPV70-hrHPV45	146	TV-TP-HPV6-hrHPV16-hrHPV18-HPV40-HPV42-HPV43-hrHPV51-HPV53-hrHPV56-hrHPV59-HPV68-HPV82
135	MG-HPV44-hrHPV56-HPV68-hrHPV16-HPV42-HPV53-HPV61-HPV82-HPV83	136	CT-TV-TP-HPV44-hrHPV56-HPV82-hrHPV59
140	CT-HPV11-HPV39-hrHPV45-HPV53-HPV69-HPV81-hrHPV31-HPV54	129	CT-NG-TV-TP-HPV11-HPV40-HPV42-hrHPV51-HPV68-hrHPV31-HPV39-hrHPV58-HPV70-HPV73-HPV52

CT: *Chlamydia trachomatis*, NG: *Neisseria gonorrhoeae*, TV: *Trichomonas vaginalis*, MG: *Mycoplasma genitalium*, hrHPV: high-risk HPV, TP: *Treponema pallidum*.

### Risk factors for any STI or CT infection in women in the Tapajós region

All HIV-infected women were on antiretroviral therapy (ART). The median HIV-1 viral loads among women with negative and positive test results for any of the four STIs were 39 (IQR, 39–39) and 39 (IQR, 39–833.75) copies/ml, respectively; the CD4 + T-cell counts were 382 (IQR, 223–567) and 416 (IQR, 217–551) cells/mm^3^, respectively. No statistically significant correlations were found (*P* = 0.286; *P* = 0.846) (S1 Table). Considering the CD4+ T-cell count as a proxy of the immunosuppression levels, Spearman's ρ test showed no significant correlation between the CD4+ T-cell count and the number of STIs (rô = -0.043, *P* = 0.790).

The identification of risk factors for STIs was determined by univariate and multivariate analyses. In the multivariate analysis, all variables were adjusted, and in the last model, the following variables remained in the model as risk factors: age ≤ 25 years increased the chance of having any of the four STIs by 5.6 (aOR = 5.60, 95% CI 2.22–14.13) and being employed or a student (aOR = 2.17, 95% CI 0.99–4.78), reporting a history of an STI (aOR = 2.37, 95% CI 1.01–5.55) and having a positive HPV DNA test (aOR = 2.82, 95% CI 1.27–6.25) significantly increased the risk of having any of the four STIs (Table 5). The multivariable analysis showed the following variables as risk factors for having a CT infection (*P* ≤ 0.05): age ≤ 25 years (aOR = 4.04, 95% CI 1.58–10.35), single status (aOR = 2.86, 95% CI 1.20–6.82) and alcohol use (aOR = 2.34, 95% CI 0.97–5.65) (Table 5).

**Table 5:** Univariate and multivariate analyses of risk factors for any STI or CT infection in HIV-uninfected and HIV-infected women.

		Any of the four STIs				CT infection			
Variables	Total (153)	N (%)	*P*-value	OR (95% CI)	Adjusted OR (95% CI)	N (%)	*P*-value	OR (95% CI)	Adjusted OR (95% CI)
**HIV status**			0.470				0.889		
HIV-uninfected	112	34 (30.4)		-	-	23 (20.5)		-	-
HIV-infected	41	10 (24.4)		-	-	08 (19.5)		-	-
**Age (years)**			<0.001				0.005		
≤25 years	31	18 (58.1)		5.06 (2.20–11.66)	5.60 (2.22–14.13)	12 (38.7)		3.39 (1.42–8.12)	4.04 (1.58–0.35)
26+	121	26 (21.5)		1.00	1.00	19 (15.7)		1.00	1.00
**Employment status**			0.027				0.222		
Unemployed	84	18 (21.4)		1.00	1.00	14 (16.7)		-	-
Employed or student	69	26 (37.7)		2.22 (1.09–4.52)	2.17 (0.99–4.78)	17 (24.6)		-	-
**Marital status**			0.085				0.017		
Single	59	22 (37.3)		1.87 (0.91–3.81)	-	18 (30.5)		2.63 (1.18–5.91)	2.86 (1.20–6.82)
Married/ living together	91	22 (24.2)		1.00	-	13 (14.3)		1.00	1.00
**Education (years)**			0.503				1.000		
Up to 17	129	36 (27.9)		-	-	27 (20.9)		-	-
18+	23	08 (34.8)		-	-	04 (17.4)		-	-
**Number of pregnancies**			0.001				0.015		
Up to 1	39	19 (48.7)		3.62 (1.65–7.94)	-	13 (33.3)		2.81 (1.20–6.59)	-
2+	106	22 (20.8)		1.00	-	16 (15.1)		1.00	-
**Hormonal contraception**			0.825				0.572		
No	67	20 (29.9)		-	-	14 (20.9)		-	-
Yes	46	14 (30.4)		-	-	11 (23.9)		-	-
No information	40	10 (25.0)		-	-	06 (15.0)		-	-
**History of cancer**			0.550				0.707		
No	106	35 (33.0)		-	-	24 (22.6)		-	-
Yes	26	07 (26.9)		-	-	5 (19.2)		-	-
**Alcohol use**			0.242				0.029		
No	106	27 (25.5)		-	-	16 (15.1)		1.00	1.00
Yes	46	16 (34.8)		-	-	14 (30.4)		2.46 (1.08–5.60)	2.34 (0.97–5.65)
**Tobacco use**			0.851				0.777		
No	130	38 (29.2)		-	-	26 (20.0)		-	-
Yes	22	06 (27.3)		-	-	05 (22.7)		-	-
**Age of first sexual intercourse (y. o.)**			0.265				0.227		
Up to 17	104	33 (31.7)		-	-	24 (23.1)		-	-
18+	48	11 (22.9)		-	-	07 (14.6)		-	-
**Lifetime number sex partners**			0.585				0.870		
1–3	68	21 (30.9)		-	-	14 (20.6)		-	-
4+	82	22 (26.8)		-	-	16 (19.5)		-	-
**Regular pap smear**			0.012				0.140		
No	20	11 (55.0)		3.31 (1.25–8.76)	-	07 (35.0)		2.28 (0.83–6.37)	-
Yes	115	31 (27.0)		1.00	-	22 (19.1)		1.00	-
**Condoms use**			0.909				0.957		
No	127	37 (29.1)		-	-	26 (20.5)		-	-
Yes	25	07 (28.0)		-	-	05 (20.0)		-	-
**History of STI**			0.162				0.120		
No	105	27 (25.7)		1.00	1.00	18 (17.1)		1.00	-
Yes	46	17 (37.0)		1.69 (0.81–3.56)	2.37 (1.01–5.55)	13 (28.3)		1.90 (0.84–4.32)	-
**Pap smear result**			0.210				-*		
Negative	119	31 (26.1)		-	-	21 (17.6)		1.00	-
Inflammation (BV)	29	11 (37.9)		-	-	10 (34.5)		2.46 (1.00–6.04)	-
LSIL	3	02 (66.7)		-	-	0 (0.0)		-	-
HSIL	2	0 (0.0)		-	-	0 (0.0)		-	-
**HPV test**			0.004				0.008		-
Negative	77	14 (18.2)		1.00	1.00	09 (11.7)		1.00	-
Positive	76	30 (39.5)		2.94 (1.40–6.15)	2.82 (1.27–6.27)	22 (28.9)		3.08 (1.31–7.23)	-

*Since the number of CT-infected women in the LSIL and HSIL categories was zero, the *P-*value could not be calculated; since the number of *Mycoplasma genitalium*-infected women in the HSIL category was zero, the *P-*value could not be calculated.

CT: *Chlamydia trachomatis*, OR: odds ratio, CI: confidence interval, aOR: adjusted odds ratio, BV: bacterial vaginosis, LSIL: low-grade squamous intraepithelial lesion, HSIL: high-grade squamous intraepithelial lesion.

## Discussion

This is the first study of STI prevalence detected by molecular testing in self-collected samples compared to clinician-collected samples in women living in the Tapajós region, Amazon, Brazil. In the present study, a high STI prevalence was identified, namely, 30.4% in HIV-uninfected women and 24.4% in HIV-infected women, as assessed by positivity in at least one of the three types of clinical specimens. This prevalence is much higher than the 2.1% detected in a previous multicenter study with HIV-infected women [5] and the 13.6% of women in the general population in Peru [18], but the prevalence was less than the 36.5% observed in HIV-infected pregnant women in northeastern Brazil [6] and the 60.6% observed in female sex workers in Peru [18]. In this study, we found an overall prevalence of STIs slightly higher in HIV-uninfected women than in HIV-infected women, although this difference was not statistically significant. We believe that this can be explained by the differences in sample size distribution between the groups, which is a limitation of our study and is discussed more below. However, we believe that the overall prevalence of STIs in HIV-infected women should be higher because these women had a higher diversity of STIs, coinfections and multiple infections, and the seroprevalence of syphilis was also high in this study.

In our study, the HIV-uninfected and HIV-infected women were able to easily understand how to perform adequate self-collection, considering the substantial and significant overall agreement of detection for each STI compared to the cervical scraping (Table 3). This finding is in agreement with results of studies conducted in other countries, where the self-collected samples were found to be a valid and reliable method for STI testing in women, with high sensitivity and high specificity and moderate to substantial agreement in comparison with clinician-collected samples [19–22]. In Brazil, we found only two studies reporting self-collected samples for STI detection performed with women in São Paulo but with different designs and objectives. In a 2006 study focusing on low-income women (n = 818), the objective was to compare the immediate response rate to the acceptability of self-collected vaginal swabs for CT, NG and TV DNA detection and rapid self-test for trichomoniasis in women participating in home-based screening and women in clinic-based screening; that is, they basically assessed the same techniques in two groups of different women. The home group had 80%, the clinic group had 76% of the immediate response rate, and no significant differences were observed in the overall prevalence of STI between groups [23]. The second study was in 2013, as part of the study previously cited, with women (n = 625) also participating in home-and clinic-based screenings that self-collected two vaginal swabs, aiming to evaluate specificity and sensitivity of the rapid self-test for trichomoniasis in both settings, having a PCR as the gold standard [24]. In spite of the importance of the scientific data reported in both studies, there is a comparative limitation because in those two studies, there was no analysis of agreement of STI detection between self-collected and clinician collected samples of the same woman and with the two specimens collected at the same time, as in our study; this approach allowed us to state that women routinely attending health clinics, either HIV-infected women or HIV-uninfected women that often do not attend or infrequently attend health clinics, were able to perform a self-collection as well as clinician-collected samples. To the best of our knowledge, this is a pioneering study conducted in Brazil on the prevalence of STI, including unpublished data on MG prevalence in the Brazilian Amazon, and comparative performance of the molecular detection of STI using scientifically validated methods among self-collected and clinician-collected samples produced by the same woman and at the same time. We preserved the scenario of care in the public health units of the region to make it more feasible to include this methodology in the screening of STI in the reality of the most vulnerable women. The unavailability of laboratory STI testing and inaccessible medical appointments were barriers to the early diagnosis of STIs of bacterial etiology that have not been well explored; however, this can be improved by less invasive and patient-centered methods, such as cervico-vaginal self-collection, which has proven to be an efficient alternative, not only for STI screening but also for cervical cancer screening [20,25].

Anogenital CT was the most common STI detected, with an overall prevalence of 20.5% for HIV-uninfected women and 19.5% for HIV-infected women. The prevalence of anogenital CT infection was higher than that found in multicenter and isolated studies conducted with HIV-uninfected and HIV-infected women in other low- and middle-income countries [26], but variable findings exist in previous studies from Brazil [5,27,28]. In the Brazilian Amazon, the prevalence of genital infection by CT ranges from ~4.8% to ~11.0% in nonindigenous women [29–31]. It is believed that, in the Tapajós region, the prevalence of CT is high in HIV-infected and HIV-uninfected women. This hypothesis is reinforced by a high seroprevalence of specific serotypes of CT (48.1%) detected in blood samples from women in Santarém, Pará [32].

Anogenital prevalence of NG identified in the two groups of participants (2.7% vs 4.9%) is in agreement with the range of 0.3% to 0.9% of NG reported for women in Brazil [5,31]; however, the prevalence of TV identified in our study (0.9 vs 9.8%) was smaller than the 18.04% identified by another study in Amazonian women [29].

The seroprevalence of syphilis (9.8%) observed in HIV-infected women was higher than that in high-risk populations for STIs in eastern Africa (8.4%) [3]. The magnitude of syphilis worldwide has been challenging to address and is a source of great concern among health authorities since the number of cases has increased dramatically and the risk of mother-to-child transmission of HIV has become an emerging threat [1,33]. Efforts have been made to improve the coverage of rapid diagnostic testing in pregnant women at antenatal care clinics in Brazil [34]. Additionally, trials for the implementation of point-of-care syphilis testing have been performed with women living in high-risk areas in the Brazilian Amazon [35].

There have been very few studies of MG prevalence, and those have assessed specific groups of women [36]. Our findings provide the first report of anogenital MG infection in northern Brazil. We identified a prevalence of 10.7% of MG infection in HIV-uninfected women without cervical lesions but with inflammation due to bacterial vaginosis, compared to 2.4% in HIV-infected women with cervical dysplasia and inflammation. This prevalence was lower than that detected in 28.1% of women living in the northeastern region of Brazil [37]. This finding was notable because HIV-uninfected women had a higher prevalence of MG infection and a total of 30.7% of coinfection and multiple infections with MG, while HIV-infected women had only 10% prevalence of coinfection and multiple infections with MG. Further epidemiological studies are needed to understand MG infection in women living in the Tapajós region.

There was no significant correlation in the univariate or multivariate analysis of HIV infection status and STI positivity and no statistically significant difference between the groups. It is important to note that all HIV-infected women in this study were on cART, and most of them were clinically and immunologically well, with virological suppression. Fortunately, this can be attributed to the efficient public health strategies implemented in Brazil to combat HIV/AIDS, such as the provision of regular medical appointments and treatment for HIV free-of-charge by the Brazilian Ministry of Health [38,39]. This study suggests that both HIV-infected and HIV-uninfected women in the Tapajós region have a high prevalence of STIs with coinfections.

Interestingly, in all cases of coinfections, there was an HPV infection; a statistically significant difference and association in the uni- and multi-variate analyses were observed, suggesting that women with HPV infection are at an increased risk of having another STI. Other studies have shown an association between HPV and other STIs and the development of cervical and anal lesions, with an increased risk for HPV-CT and HPV-MG [40–42].

One limitation of our study was the small sample size in the group of HIV-infected women, which may have been influenced by difficulties with accessibility and the infrastructure of the health units in Santarém, as well as the lack of data in the literature on HIV and STI prevalence in the population studied. To maintain homogeneity between the two groups of women, we decided to match them based on age and the date of diagnosis and survey. We established the criterion of including only HIV-infected women who had been diagnosed with HIV infection during the same study period and in the same age groups as HIV-uninfected women (36.9 ± 13.1). The WHO recognizes that one of the current challenges of STI control strategies is to improve the quality of future estimates and supporting countries to generate their own national estimates. This should be done with more data from studies conducted on the general population with low risk and on key populations. WHO is also exploring alternative methods for generating STI prevalence and incidence estimates, including approaches that use data collected from studies conducted in key populations and the use of new technologies to facilitate diagnosis [33,43]. The strength of this study is that the unpublished epidemiological findings of the prevalence of STIs in a population of HIV-infected and HIV-uninfected women and the significant agreement in the STI detection in self-collected compared to clinician-collected samples obtained meet the needs noted by the WHO. Accurate estimates are crucial for guiding broader STI prevention and control efforts, primarily in limited-resource settings at higher risk and with very little scientific data available. The methods used in this study, as well as the implications of the findings, may be applied to new studies conducted among similar populations in isolated and low-resource settings. Further epidemiological studies are needed to elucidate STIs, with attention to the ascent of MG infection due to the increase in the prevalence and the high capacity of resistance aggravated by the lack of knowledge of the population and the unfamiliarity of health professionals.

STI surveillance strategies in the Tapajós region should be targeted to the general population of women, independent of HIV-infected status, with a focus on young women and adolescents with a history of STI or HPV infection who are single and use alcohol, especially during their reproductive life. The risk factors found are well established in the literature [27,28,40,41]. Cross-sectional population-based studies indicate that not only geographic but also political isolation are responsible for unequal access to health services and maternal and child care in more urbanized areas of the Amazon [44,45]. The Tapajós region is geographically isolated, and people have difficulty accessing the public health units in peripheral areas of Santarém.

Efforts should aim to reduce the burden of STIs for all women, especially at a time when cART reduces the burden of HIV infection, and women can have the ability to talk more about reproductive choices. The mobilization of resources for the implementation of cervico-vaginal self-collection as an alternative for the detection of STIs by rapid diagnostic tests (RDTs) based on nucleic acid amplification assays (NAATs) should be seriously evaluated as a strategy to improve screening programs [33], considering the insufficient coverage of neglected women living in isolated areas with high incidence and mortality of cervical cancer and high prevalence of STI, as in the Tapajós region.

## Conclusions

In summary, we identified a high prevalence of STIs and a significant positive agreement rate between self-collected and clinician-collected samples in this study. Considering these findings, we suggest the implementation of STI screening and prevention control programs in groups at high risk of acquiring and transmitting STIs, such as young women and HIV-infected women, especially in resource-limited settings and isolated regions of the country. In addition, further epidemiological studies are warranted to investigate the role of HPV with other STIs and the impact of CT and MG on HIV-infected and HIV-uninfected women living in the Tapajós region, Amazon, and other regions of Brazil.

## Supporting information

S1 Table The Chi-square test for categorical variables and Mann-Whitney U test for continuous variables, both at 95% CI and *p* value ≤ 0.05. (DOCX)
