# Supplementary material for: Sexually transmitted infections among HIV-infected and HIV-uninfected women in the Tapajós region, Amazon, Brazil: Self-collected vs. clinician-collected samples
Source: PLoS One. 2019 Apr 23;14(4):e0215001. doi: 10.1371/journal.pone.0215001 (PMC6478302; doi:10.1371/journal.pone.0215001)
Supplement: S1 Table — (DOCX) [file pone.0215001.s001.docx]

| **S1 Table. The Chi-square test for categorical variables and Mann-Whitney U test for continuous variables, both at 95% CI and *p* value ≤ 0.05.** | | | |
| --- | --- | --- | --- |
| **Variables** | **Negative for STI**  **(n = 31)** | **Positive for STI**  **(n = 10)** | ***p* value** |
| HIV Viral load |  |  | 0.222 |
| <40 copies/mL | 25 (80.6%) | 06 (60.0%) |  |
| ≥40 copies/mL | 06 (19.4%) | 04 (40.0%) |  |
| CD4+ T-cell counts |  |  | 0.991 |
| <200 cells/mm^3^ | 06 (19.4%) | 02 (20.0%) |  |
| 200-500 cells/mm^3^ | 15 (48.4%) | 05 (50.0%) |  |
| >500 cells/mm^3^ | 10 (32.3%) | 03 (30.0%) |  |
|  | **Median (IQR)** | **Median (IQR)** |  |
| HIV Viral load | 39 (39-39) | 39 (39-833.75) | 0.286 |
| CD4+ T-cell count | 382 (223-567) | 416 (217-551) | 0.846 |
